# Species Identification and Antibiotic Resistance Prediction by Analysis of Whole-Genome Sequence Data by Use of ARESdb: an Analysis of Isolates from the Unyvero Lower Respiratory Tract Infection Trial

**DOI:** 10.1128/JCM.00273-20

**Published:** 2020-06-24

**Authors:** Ines Ferreira, Stephan Beisken, Lukas Lueftinger, Thomas Weinmaier, Matthias Klein, Johannes Bacher, Robin Patel, Arndt von Haeseler, Andreas E. Posch

**Affiliations:** a Ares Genetics GmbH, Vienna, Austria; b Curetis GmbH, Holzgerlingen, Germany; c Mayo Clinic, Rochester, Minnesota, USA; d Center for Integrative Bioinformatics Vienna, Max Perutz Laboratories, University of Vienna and Medical University of Vienna, Vienna, Austria; University Hospital Münster

**Keywords:** antimicrobial resistance, whole-genome sequencing, infectious disease diagnostics, antimicrobial susceptibility testing, lower respiratory tract infection

## Abstract

Whole-genome sequencing (WGS) is now routinely performed in clinical microbiology laboratories to assess isolate relatedness. With appropriately developed analytics, the same data can be used for prediction of antimicrobial susceptibility. We assessed WGS data for identification using open-source tools and antibiotic susceptibility testing (AST) prediction using ARESdb compared to matrix-assisted laser desorption ionization-time of flight mass spectrometry (MALDI-TOF MS) identification and broth microdilution phenotypic susceptibility testing on clinical isolates from a multicenter clinical trial of the FDA-cleared Unyvero lower respiratory tract infection (LRTI) application (Curetis). For the trial, more than 2,000 patient samples were collected from intensive care units across nine hospitals and tested for LRTI. The isolate subset used in this study included 620 clinical isolates originating from 455 LRTI culture-positive patient samples. Isolates were sequenced using the Illumina Nextera XT protocol and FASTQ files with raw reads uploaded to the ARESdb cloud platform (ares-genetics.cloud; released for research use in 2020). The platform combines Ares Genetics’ proprietary database ARESdb with state-of-the-art bioinformatics tools and curated public data. For identification, WGS showed 99 and 93% concordance with MALDI-TOF MS at the genus and species levels, respectively. WGS-predicted susceptibility showed 89% categorical agreement with phenotypic susceptibility across a total of 129 species-compound pairs analyzed, with categorical agreement exceeding 90% in 78 species-compound pairs and reaching 100% in 32. Results of this study add to the growing body of literature showing that, with improvement of analytics, WGS data could be used to predict antimicrobial susceptibility.

## Abstract (precis)

Whole-genome sequencing (WGS) is now routinely performed in clinical microbiology laboratories to assess isolate relatedness. With appropriately developed analytics, the same data can be used for prediction of antimicrobial susceptibility. We assessed WGS data for identification using open-source tools and antibiotic susceptibility testing (AST) prediction using ARESdb compared to matrix-assisted laser desorption ionization-time of flight mass spectrometry (MALDI-TOF MS) identification and broth microdilution phenotypic susceptibility testing on clinical isolates from a multicenter clinical trial of the FDA-cleared Unyvero lower respiratory tract infection (LRTI) application (Curetis).

## INTRODUCTION

Antimicrobial resistance (AMR) is listed as a serious global health threat by multiple international groups (1, 2). The danger was reflected in the *Review on Antimicrobial Resistance* by O’Neill in 2014, projecting more than 10 million annual deaths by 2050 as a result of AMR (3). Bacteria have evolved multiple mechanisms to evade antibiotic treatment; use of antibiotics has contributed to the emergence and spread of multidrug-resistant bacteria (4, 5). At the same time, few novel antibiotics are in development, and prominent pharmaceutical companies have cut back their research and development efforts in the infectious diseases space (6). Appropriate patient treatment and AMR stewardship are needed to curtail the rise of multidrug-resistant bacteria, appropriately treat patients, and improve patient outcomes; AMR diagnostics may be helpful in this regard (7). Bacterial cultures and antimicrobial susceptibility testing (AST) are established tools for detecting, identifying, and defining the antimicrobial susceptibility of bacteria in clinical practice. Their advantages and disadvantages have been reviewed extensively (8, 9). In recent years, whole-genome sequencing (WGS), combined with high-performance bioinformatics, has become available and has been adopted into clinical practice, mostly for ascertaining isolate relatedness for infection prevention and control purposes. The WGS data used for typing, or clonality testing, can theoretically be used clinically for isolate identification alongside antimicrobial susceptibility prediction. Lacking, however, are broadly applicable bioinformatics platforms suitable for use in clinical laboratories and the demonstration of their performance.

Here, we compared WGS-based bacterial identification and antibacterial susceptibility testing against matrix-assisted laser desorption ionization-time of flight mass spectrometry (MALDI-TOF MS) identification and broth microdilution susceptibility testing using a set of clinical isolates from the clinical trial of the Unyvero lower respiratory tract infection (LRTI) application, a multiplex-PCR based sample-to-answer solution manufactured by Curetis. LRTI is a leading cause of death in intensive care unit (ICU) patients globally. The incidence of hospital-acquired LRTI in Europe and the United States is estimated at 1 to 12 cases per 1,000 individuals (10–12); 10 to 39% of patients receive inappropriate empirical treatment (13, 14), in part because of the spread of AMR.

## MATERIALS AND METHODS

### Microbiology culture.

A total of 483 patient samples—tracheal aspirate and bronchoalveolar lavage specimens—were cultured and polymicrobial cultures worked up by colony morphology, resulting in 664 cultured, microbial, clinical isolates. All clinical isolates were identified with a Bruker Microflex LT/SH MALDI-TOF MS Biotyper (MBT subtyping module RUO 3.1.65) and tested for antibiotic susceptibility by broth microdilution.

### Broth microdilution antimicrobial susceptibility testing.

MICs were determined once using the broth microdilution method in 96-well plates following European Committee on Antimicrobial Susceptibility Testing (EUCAST) recommendations. Testing was performed on 21 compounds spanning 10 compound classes (Table 1). Quality control isolates Escherichia coli ATCC 25922, ATCC 35218, and NCTC 13846, Pseudomonas aeruginosa ATCC 27853, Streptococcus pneumoniae ATCC 49619, Haemophilus influenzae ATCC 49766, and Staphylococcus aureus ATCC 29213 were used for the antibiotic susceptibility testing panels. Panels were considered acceptable for testing when the quality control results met the control ranges described by EUCAST.

**TABLE 1:** List of compounds and compound classes used for microdilution AST

Compound	Class
Ampicillin, benzylpenicillin, amoxicillin-clavulanic acid, piperacillin-tazobactam	Penicillins
Cefuroxime	Second-generation cephalosporins
Ceftazidime, ceftriaxone	Third-generation cephalosporins
Cefepime	Fourth-generation cephalosporins
Ertapenem, imipenem, meropenem	Carbapenems
Trimethoprim, trimethoprim-sulfamethoxazole	Folate pathway inhibitors
Ciprofloxacin, levofloxacin, moxifloxacin	Fluoroquinolones
Amikacin, gentamicin, tobramycin	Aminoglycosides
Erythromycin	Macrolides
Tetracycline	Tetracyclines

Briefly, testing panels were constructed by a commercial vendor (IHMA Europe) as follows: for members of the *Enterobacterales*, amikacin (0.25 to 32 μg/ml), amoxicillin-clavulanic acid (constant 2 mg/liter clavulanic acid) (1 to 16 μg/ml), ampicillin (1 to 16 μg/ml), cefepime (0.008 to 8 μg/ml), ceftazidime (0.03 to 8 μg/ml), ceftriaxone (0.015 to 4 μg/ml), cefuroxime (1 to 16 μg/ml), ciprofloxacin (0.002 to 1 μg/ml), ertapenem (0.002 to 2 μg/ml), gentamicin (0.12 to 8 μg/ml), imipenem (0.03 to 16 μg/ml), levofloxacin (0.004 to 2 μg/ml), meropenem (0.004 to 16 μg/ml), piperacillin-tazobactam (constant 4 mg/liter tazobactam) (0.12 to 32 μg/ml), tobramycin (0.12 to 8 μg/ml), and trimethoprim-sulfamethoxazole (1:19) (0.015 to 8 μg/ml); for P. aeruginosa, Acinetobacter baumannii, and Stenotrophomonas maltophilia, amikacin (0.5 to 32 μg/ml), cefepime (0.25 to 16 μg/ml), ceftazidime (0.5 to 32 μg/ml), ciprofloxacin (0.125 to 8 μg/ml), gentamicin (0.25 to 16 μg/ml), imipenem (0.25 to 32 μg/ml), levofloxacin (0.125 to 8 μg/ml), meropenem (0.125 to 32 μg/ml), piperacillin-tazobactam (constant 4 μg/ml tazobactam) (0.12 to 64 μg/ml), tobramycin (0.25 to 16 μg/ml), and trimethoprim-sulfamethoxazole (1:19) (0.125 to 16 μg/ml); for S. aureus, benzylpenicillin (0.03 to 2 μg/ml), ciprofloxacin (0.06 to 8 μg/ml), erythromycin (0.12 to 4 μg/ml), gentamicin (0.06 to 4 μg/ml), levofloxacin (0.03 to 8 μg/ml), moxifloxacin (0.008 to 1 μg/ml), tetracycline (0.06 to 4 μg/ml), trimethoprim (0.06 to 8 μg/ml), and trimethoprim-sulfamethoxazole (1:19) (0.008 to 8 μg/ml).

MIC values were converted into categorical resistant (R), susceptible (S), intermediate (I), and susceptible dose-dependent (SDD) phenotypes per species-compound pair according to breakpoints defined by the Clinical and Laboratory Standards Institute (CLSI) guidelines (15). EUCAST guidelines were used for *in vitro* testing, and CLSI criteria were used for interpretation because WGS AST models in ARESdb were trained on CLSI criteria (see “WGS AST model training, validation, and evaluation”). Tested dilution ranges were confirmed to include CLSI interpretive breakpoints for all species-compound pairs.

### Whole-genome sequencing.

DNA was extracted from isolate cultures using the UltraClean microbial DNA isolation kit according to the manufacturer’s protocol (Mo Bio Laboratories Inc.). Next-generation sequencing (NGS) libraries were prepared in a 96-well format using a NexteraXT DNA sample preparation kit and a NexteraXT index kit for 96 indexes (Illumina) according to the manufacturer’s protocol. Sequencing was run on Illumina NextSeq 500, v2, high output, 2 × 150 bp, with a minimum targeted depth of 30. Samples were demultiplexed and Illumina adapter residuals trimmed.

### ARESdb and computational data analysis.

Data analysis workflows were written with Nextflow v18.10.1 and implemented on the ARESdb cloud platform (ares-genetics.cloud). The platform combines Ares Genetics’ proprietary database ARESdb, an extension of the GEAR database described by Galata et al. (16), with state-of-the-art open-source tools and public data. At the time of the study, ARESdb comprised curated genotype-phenotype data for approximately 35,000 bacterial strains and more than 100 antibiotics, originating largely from GEAR as well as from freely accessible data sets, including data from PATRIC and NDARO. At the time of submission, ARESdb has further grown and contains matched whole-genome sequencing and antibiotic susceptibility data for more than 50,000 bacterial strains.

### (i) WGS data upload and ARESdb access.

Data upload, WGS AST analysis, and interactive reporting based on ARESdb were performed via ares-genetics.cloud (https://ares-genetics.cloud) hosted on Amazon Web Services (AWS), which is accessible for research use after registration. FASTQ files containing NGS raw reads from the 664 samples evaluated in this study were uploaded to the secure user space via secure file transfer protocol. Reports of predicted S/R phenotypes were subsequently downloaded and used for WGS AST performance evaluation in this study.

### (ii) WGS data quality control and genome assembly.

Illumina NextSeq 500 reads were quality checked using FastQC v0.11.6 and MultiQC v1.6 (17, 18). Platform-specific adapters were removed and paired-end reads trimmed using Trimmomatic v0.38 (19). Reads were deduplicated prior to downstream analysis with FastUniq v1.1 (20). Sequenced isolates were *de novo* assembled with SPAdes v3.12.0 and annotated with Prokka v1.13 prior to quality control through Quast v4.6.3 and assembly coverage determination with BWA v0.7.17 and BEDTools v.2.27.1 for indexing and alignment and Samtools v1.9 for sorting the BAM files (21–26).

CheckM was used to evaluate assembly quality and contamination (27). Isolates that had less than 90% completeness, more than 10% contamination as per CheckM, or a quality measure of less than 50% were considered contaminated or low quality and were consequently removed. The quality measure was calculated as the completeness CheckM metric minus five times the contamination as in reference 28. From the 664 initial isolates, 44 were removed that failed to meet the defined quality criteria. Of the remaining 620 isolates, 25 presented a genome bigger than expected for the identified species. For these 25, after manual inspection of the assembly files, a long tail of small contig fragments (below 300 bp) with a larger GC content than that of the main genome was observed. To try to rescue as many isolates as possible, any contig with a length below 300 bp was removed.

### (iii) WGS-based identification.

WGS-based *in silico* identifications were determined from sequencing reads with Kraken v1.0 using Kraken’s “full kraken database” based on the NCBI nonredundant database (29). Assigned taxonomy identifications were double-checked using contig assembly similarities calculated by SourMash 2.0.0a using the NCBI RefSeq database (30). Identifications were compared for agreement on the species and genus rank per isolate culture between MALDI-TOF MS and WGS-based *in silico* identification.

### (iv) WGS AST model training, validation, and evaluation.

WGS AST classification models for prediction of antibiotic susceptibility/resistance (S/R) were trained per species-compound pair using ARESdb. In summary, a total of 129 independent WGS AST models covering 21 antibiotic compounds and 13 pathogenic species were assessed in this study.

For each of the 13 species, an exhaustive 15-mer count matrix was built based on *de novo* genome assemblies from ARESdb using KMC 3.1.0 (31). For each 15-mer count matrix, zero-variance 15-mers were removed and the top 50,000,000 15-mers with the highest variance were selected. All subsequent feature selection and model training procedures were performed independently for each of the 129 species-compound combinations assessed.

For feature selection and model training, the 15-mer count matrix of a given species was randomly split into training/holdout subsets by the ratio 80% to 20%. Stratified splitting was performed based on the MIC of a given compound, thereby ensuring homogenous distribution of resistance phenotypes in training and holdout subsets. Training/holdout 15-mer count matrices were subsequently binarized to yield presence/absence matrices of genome assemblies and 15-mers. Resistance phenotypes as measured by the MIC were subsequently binarized to derive R/S class labels. Intermediate and susceptible dose-dependent phenotypes were treated as susceptible. For each 15-mer in the training subset, the presence/absence pattern was tested for association with the resistance phenotype using the χ^2^ test as implemented in scikit-learn (32). The 15-mers were then filtered for *P* values of <0.05 prior to inclusion in subsequent model training. Additionally, the feature space for each species-compound combination was expanded to include all 15-mers mapping to curated AMR markers that have been either described in scientific studies or identified by our recently published high-throughput biomarker validation procedure (33). The method was thus designed to select features that are either strongly correlated with the phenotype or known and functionally meaningful AMR markers.

Using the selected features, extreme gradient boosting binary classifiers were trained for the prediction of S/R categories with XGBoost 0.82 (34). Hyperparameters for model training were optimized for balanced accuracy using random search over a defined variable space and validated by 5-fold cross-validation. Finally, optimized models were further validated on the retained holdout subset confirming generalizability before being evaluated for WGS-based AST on isolates from the Unyvero LRTI trial.

### (v) Comparison of microdilution AST and WGS AST.

Observed resistant and susceptible phenotypes from microdilution AST were compared to WGS-based *in silico*-predicted AST phenotypes per compound-isolate pair. The comparison was performed using binary classification metrics such as categorical agreement, as well as major errors (MEs) and very major errors (VMEs), referring to false-resistant (false-positive) and false-susceptible (false-negative) predictions, respectively.

### Data availability.

Raw sequence reads and genome assemblies are available from the NCBI BioProject repository under project no. PRJNA553678, “Clinical Utility of Antibiotic Susceptibility Testing by Whole Genome Sequencing.”

## RESULTS

### Study design.

**FIG 1:**
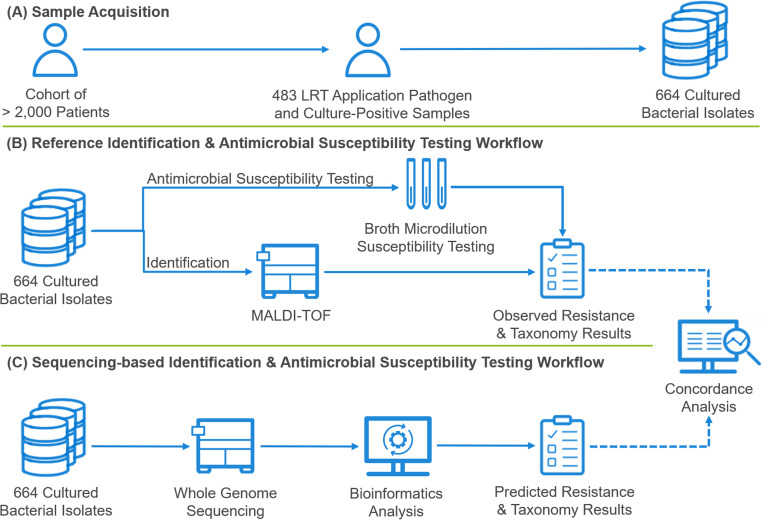
Overview of the sample acquisition, identification, and antimicrobial susceptibility testing workflows. (A) A total of 664 cultured isolates from 483 patient samples which were positive for at least one Unyvero LRTI application pathogen and which were culture positive, out of a cohort of more than 2,000 patient samples (tracheal aspirate and bronchoalveolar lavage specimens combined), were selected for study. (B and C) Isolates were identified by MALDI-TOF MS and *in vitro* antimicrobial susceptibility testing performed using broth microdilution (B), as well as being subjected to WGS-based identification using open-source tools and antimicrobial susceptibility prediction using ARESdb (C).

More than 2,000 patient samples were tested for LRTI during the trial. A total of 664 isolates from 483 samples that were culture positive for at least one Unyvero LRTI application pathogen (a subset of the overall culture-positive specimens) were selected for this study. Isolates were subjected to identification via matrix-assisted laser desorption ionization time-of-flight mass spectrometry (MALDI-TOF MS) and phenotypic susceptibility testing by broth microdilution in parallel to WGS and subsequent WGS-based identification using open-source tools and AST prediction using ARESdb (Fig. 1). Concordance between WGS-based identification and MALDI-TOF MS was assessed at the species and genus levels. For antimicrobial susceptibility assessment, concordance was evaluated by comparing results of broth microdilution testing to predicted susceptibility using WGS data.

### WGS quality control.

Assembly depth ranged from 43 to 453, with a mean of 141. WGS data and *de novo* assemblies were quality checked and contaminated assemblies excluded (see Materials and Methods). Contaminated isolates were identified using CheckM following the method described by Parks et al. (27) and removed from the study. After removal of 44 isolates due to sequence contamination, the final test set contained 620 isolates.

### Comparison of MALDI-TOF MS and WGS taxonomy results.

MALDI-TOF MS and WGS-based identification agreed at the species and genus levels for 93 and 99% of isolates, respectively. Four isolates (0.6%) were discrepant at the genus level (see Table S1 in the supplemental material). MALDI-TOF MS identified 28 species (see Fig. S1) in contrast to 30 identified via WGS (see Table S2). The analysis covered the following genera: Acinetobacter, *Citrobacter*, *Enterobacter*, *Escherichia*, *Haemophilus*, *Klebsiella*, *Moraxella*, *Morganella*, *Proteus*, *Providencia*, *Pseudomonas*, *Raoultella*, *Serratia*, *Staphylococcus*, *Stenotrophomonas*, and *Streptococcus*. The four isolates discrepant at the genus level were excluded from the comparison between broth microdilution testing and WGS susceptibility predictions.

### Comparison of broth microdilution AST and WGS-based prediction of antimicrobial susceptibility.

**FIG 2:**
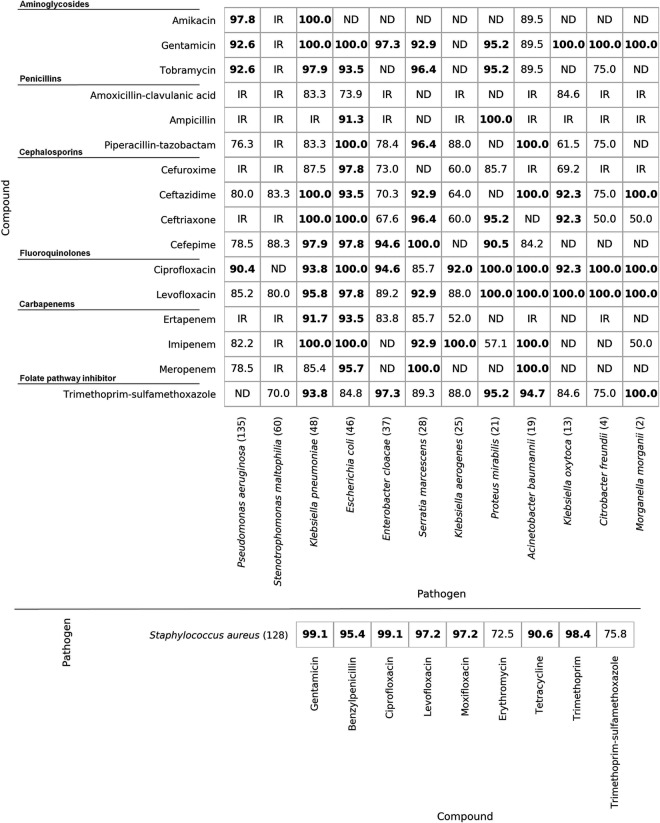
Percent categorical agreement per species-compound pair, with Gram-negative bacteria shown on the top and S. aureus on the bottom. Species considered intrinsically resistant to a given compound according to CLSI guidelines (15) are labeled “IR.” Species-compound pairs which were not tested with broth microdilution AST or for which no WGS susceptibility prediction was run are labeled “ND.” Species sample counts are in parentheses. Categorical agreement values of >90% are in bold.

Broth microdilution AST results were compared to WGS-predicted susceptibility and analyzed for agreement as to the determined susceptible (S) or resistant (R) phenotypes according to CLSI interpretive guidelines. Intermediate (I) and susceptible dose-dependent (SDD) phenotypes were treated as susceptible. Susceptible and resistant phenotypes were determined per isolate-compound pair. From the 616 isolates that passed quality control and had concordant genus ID results, WGS susceptibility predictions were obtained using Ares Genetics’ proprietary database ARESdb (see Materials and Methods) for species-compound pairs present in the database. Comparison between broth microdilution and WGS-predicted AST was performed on the 566 isolates for which data from the given methods was available for at least one compound. There was 89% categorical agreement between WGS-predicted AST and broth microdilution AST. One-on-one comparisons were performed by species and compound pairs if that pair was tested via broth microdilution AST and WGS-predicted susceptibility was available. For 78 of 129 compared pairs, categorical agreement was ≥90%, with 32 pairs showing 100% categorical agreement (Fig. 2). Overall, a major error (ME) rate of 8% and very major error (VME) rate of 19% were calculated across all isolate-compound pairs. VME and ME rates for all species-compound pairs are shown in Fig. S2 and S3. Over half of the species-compound pairs had MEs of ≤2.5%, with notable exceptions being cephalosporins for Klebsiella aerogenes and Enterobacter cloacae, which had ME rates as high as 76.9%.

When data were grouped by compound class, categorical agreement ranged from 72.5% for macrolides to 95.8% for aminoglycosides. All compound classes except for macrolides presented categorical agreement above 80% (Table 2).

**TABLE 2:** Summary of categorical agreement, very major errors, and major errors per compound class for WGS-based susceptibility prediction in comparison with broth microdilution susceptibility testing^*a*^

Compound class	No. of compounds	No. of isolates	Categorical agreement (%)	VME (%)	ME (%)	TP	TN	FP	FN
Aminoglycosides	3	462	924/965 (95.8)	19/115 (16.5)	22/850 (2.6)	96	828	22	19
Fluoroquinolones	3	547	1067/1143 (93.4)	34/382 (8.9)	42/761 (5.5)	348	719	42	34
Tetracyclines	1	128	116/128 (90.6)	2/5 (40.0)	10/123 (8.1)	3	113	10	2
Macrolides	1	109	79/109 (72.5)	26/69 (37.7)	4/40 (10.0)	43	36	4	26
Folate pathway inhibitors	2	431	486/559 (86.9)	39/106 (36.8)	34/453 (7.5)	67	419	34	39
Penicillins	4	485	549/638 (86.1)	38/217 (17.5)	51/421 (12.1)	179	370	51	38
Cephalosporins (second generation)	1	190	156/190 (82.1)	9/68 (13.2)	25/122 (20.5)	59	97	25	9
Cephalosporins (third generation)	2	438	549/641 (85.6)	44/187 (23.5)	48/454 (10.6)	143	406	48	44
Cephalosporins (fourth generation)	1	394	349/394 (88.6)	18/104 (17.3)	27/290 (9.3)	86	263	27	18
Carbapenems	3	361	681/784 (86.9)	34/139 (24.5)	69/645 (10.7)	105	576	69	34

a Categorical agreement, very major errors (VME), and major errors (ME) were calculated from the overall number of true positives (TP), true negatives (TN), false positives (FP), and false negatives (FN) obtained per compound class.

Categorical agreement per species ranged from 76.9% (K. aerogenes) to 95.2% (Acinetobacter baumannii). Categorical agreement was >80% for Stenotrophomonas maltophilia, Citrobacter freundii, Enterobacter cloacae, Pseudomonas aeruginosa, Morganella morganii, Klebsiella oxytoca, Proteus mirabilis, Staphylococcus aureus, Serratia marcescens, Klebsiella pneumoniae, Escherichia coli, and A. baumannii (Table 3).

**TABLE 3:** Summary of categorical agreement, very major errors, and major errors per species WGS-based susceptibility prediction in comparison with broth microdilution^*a*^

Species	No. of compounds	No. of isolates	Categorical agreement (%)	VME (%)	ME (%)	TP	TN	FP	FN
Acinetobacter baumannii	11	19	199/209 (95.2)	5/146 (3.4)	5/63 (7.9)	141	58	5	5
Escherichia coli	15	46	653/690 (94.6)	26/143 (18.2)	11/547 (2.0)	117	536	11	26
Klebsiella pneumoniae	15	48	677/720 (94.0)	13/110 (11.8)	30/610 (4.9)	97	580	30	13
Serratia marcescens	12	28	314/336 (93.5)	11/26 (42.3)	11/310 (3.5)	15	299	11	11
Staphylococcus aureus	9	128	950/1038 (91.5)	37/341 (10.9)	51/697 (7.3)	304	646	51	37
Proteus mirabilis	10	21	192/210 (91.4)	5/52 (9.6)	13/158 (8.2)	47	145	13	5
Klebsiella oxytoca	9	13	101/117 (86.3)	7/20 (35.0)	9/97 (9.3)	13	88	9	7
Morganella morganii	7	2	12/14 (85.7)	2/10 (20.0)	0/4 (0.0)	8	4	0	2
Pseudomonas aeruginosa	10	135	1153/1350 (85.4)	84/294 (28.6)	113/1056 (10.7)	210	943	113	84
Enterobacter cloacae	10	37	313/370 (84.6)	21/79 (26.6)	36/291 (12.4)	58	255	36	21
Citrobacter freundii	8	4	26/32 (81.3)	4/5 (80.0)	2/27 (7.4)	1	25	2	4
Stenotrophomonas maltophilia	4	60	193/240 (80.4)	35/123 (28.5)	12/117 (10.3)	88	105	12	35
Klebsiella aerogenes	9	25	173/225 (76.9)	13/43 (30.2)	39/182 (21.4)	30	143	39	13

a Categorical agreement, very major errors, and major errors were calculated from the overall number of true positives (TP), true negatives (TN), false positives (FP), and false negatives (FN) obtained per species. Data are sorted by categorical agreement in descending order.

ESKAPE pathogens (A. baumannii, K. pneumoniae, S. aureus, E. cloacae, and P. aeruginosa), representing 5 of the 13 species analyzed, and E. coli were selected to illustrate species-level results observed for WGS-based susceptibility prediction, as shown in the species-compound results.

For A. baumannii, WGS-based susceptibility prediction had >84% categorical agreement, with 0 VMEs for piperacillin-tazobactam, ceftazidime, cefepime, ciprofloxacin, levofloxacin, imipenem, meropenem, and trimethoprim-sulfamethoxazole (see Fig. S2). For aminoglycosides, the VME rate ranged from 7.7 to 18.2%. The ME rate was 0% for all compounds except gentamicin (ME = 16.7%), cefepime (ME = 37.5%), and trimethoprim-sulfamethoxazole (ME = 20.0%) (see Fig. S3).

For E. coli, categorical agreement was >90% except for amoxicillin-clavulanic acid (73.9%) and trimethoprim-sulfamethoxazole (84.8%). VMEs for E. coli varied between compounds, ranging from 0% for gentamicin, tobramycin, ceftriaxone, cefepime, and ciprofloxacin to 100% for ertapenem, while MEs ranged mostly from 0 to 3.6% except for meropenem (ME = 4.3%), ceftazidime (ME = 5.1%), tobramycin (ME = 7.0%), and ampicillin (ME = 8.3%).

Categorical agreement for K. pneumoniae was above 83% for all compounds. VMEs varied from 0% for amikacin, gentamicin, tobramycin, cefuroxime, ceftazidime, ceftriaxone, cefepime, ertapenem, imipenem, and meropenem to 43.8% for amoxicillin-clavulanic acid. The ME rate was between 0 and 5.3% for all compounds except for piperacillin-tazobactam (16.7%), cefuroxime (15.8%), ertapenem (8.7%), and meropenem (15.2%).

For P. aeruginosa, categorical agreement for all compounds was above 76%. VMEs varied from 10% for ciprofloxacin to 66.7% for amikacin. The ME rate varied from 0.8% for amikacin to 21.6% for meropenem.

S. aureus showed categorical agreement of >90% for all compounds except erythromycin (72.5%) and trimethoprim-sulfamethoxazole (75.8%). VMEs varied from 0% for fluoroquinolones to 50.0% for gentamicin and trimethoprim-sulfamethoxazole; MEs varied from 0 to 22.5%.

A. baumannii, P. aeruginosa, and *Enterobacter* species resistant to carbapenems and *Enterobacter* species resistant to third-generation cephalosporins are in the most severe, critical category of the WHO priority list (35, 36). For A. baumannii, categorical agreement for carbapenem susceptibility prediction was 100%. For P. aeruginosa, categorical agreement for carbapenem susceptibility prediction was between 78.5 and 82.2%; for E. cloacae, categorical agreement was 83.8% for ertapenem. No WGS AST models were available for meropenem and imipenem. Categorical agreement for E. cloacae susceptibility prediction for third-generation cephalosporins ranged from 67.6 to 70.3%.

## DISCUSSION

### Bacterial identification by WGS.

MALDI-TOF MS and WGS-based identification agreed for 93% of isolates at the species level and 99% at the genus level. Agreement in previous reports has varied from 77.6% to 100% at the species level, depending on the species and genus analyzed (37–43). There were 4 isolates with identifications that were discrepant at the genus level. In 3 of these cases, the isolates originated from polymicrobial cultures; discordance at the species and especially the genus level may have arisen from incomplete workup of polymicrobial samples during culture. Since discrepancies between MALDI-TOF MS and WGS-based identification are unlikely at the genus level, experimental error was assumed, and the 4 affected isolates were excluded from further analysis.

From an initial 483 patient samples, 664 bacterial isolates were obtained, indicating a high detection rate of polymicrobial lower respiratory tract microbiota in multiple cases. The rate of polymicrobial respiratory tract infections found in previous studies ranged from 11% to 28% (13, 44, 45). While this study focused on the evaluation of WGS-predicted AST for isolates, further development of sequencing applicable to polymicrobial infections with predictive AST directly from patient specimens is needed.

### Comparison of microdilution AST and WGS AST.

Overall achieved categorical agreement of WGS AST was 89%, with an ME rate of 8% and a VME rate of 19%. Categorical agreement per compound class was lowest for macrolides. Erythromycin, the only macrolide included, showed a categorical agreement of 72.5% in S. aureus. Known resistance mechanisms include ribosomal modification by methylases, mutations in rRNA, and compound transport via efflux pumps (46). Efflux pumps can be subdivided into multiple classes, some of which are nonspecific with regard to their substrates and can act as general drug efflux pumps, which are tightly regulated and can cause false-positive (false-resistant) results (46).

A principal limitation of genotypic methods for susceptibility prediction is the possible disconnect between the presence of a genetic marker and its expression. False positives (MEs) are expected for genetic markers that are tightly regulated, like efflux pumps or inducible β-lactamases, such as that encoded by *ampC*.

For the second-generation cephalosporins, cefuroxime showed an overall categorical agreement of 82.1%. Categorical agreement was particularly low for K. aerogenes and K. oxytoca. Ceftazidime and ceftriaxone, both third-generation cephalosporins, followed, with an overall categorical agreement of 85.6%. For these compounds, low categorical agreement was mainly observed with C. freundii, M. morganii, K. aerogenes, and E. cloacae.

Overall categorical agreement for the β-lactam–β-lactamase inhibitor combinations amoxicillin-clavulanate and piperacillin-tazobactam was 82.7%. The inhibitory action of the β-lactamase inhibitor is challenging to predict using genomics, making β-lactam–β-lactamase inhibitor combinations prone to high ME rates.

Across compounds, A. baumannii performed best at 95.2% categorical agreement, with 5 VMEs (3.4%) and 5 MEs (7.9%). Lowered predictive performance for P. aeruginosa with 84 VMEs (28.6%) may be due to complex regulation mechanisms present in this species (47). WGS prediction of AST for K. aerogenes, M. morganii, and C. freundii also suffered from high VMEs. The results obtained for K. aerogenes can be explained by the lack of comprehensive studies on antimicrobial resistance in this species. Passarelli‐Araujo et al. reported in 2019 on the genetic diversity and the structure of the population, as well as the resistance genes encoded in the core genome (48). With regard to M. morganii and C. freundii, only 6 isolates representing these 2 species were analyzed, indicating a low prevalence and resulting in challenges in obtaining data from these species.

To achieve regulatory approval of a new AMR diagnostic test, the FDA stipulates that categorical agreement should be >90%, VME rates should be <1.5%, and ME rates should be <3% (8). Of all 129 species-compound pairs, 78 met the FDA criteria for categorical agreement and 63 and 65 met the FDA criteria for VME and ME, respectively. In 36 of 129 pathogen-drug pairs, WGS prediction of AST categorical agreement, VME, and ME were in line with FDA guidelines. As WGS AST prediction algorithms evaluated in this study have been optimized for (balanced) accuracy based on matched genotype-phenotype training data in ARESdb, it is not surprising that the FDA accuracy acceptance categorical agreement criterion was met for a larger fraction of species-compound pairs than the VME and ME criteria. To further improve performance, improvement in WGS prediction of AST by optimizing prediction algorithms specifically for FDA acceptance criteria is required, as well as incorporation of intermediate and susceptible dose-dependent phenotypes into these algorithms. Here, intermediate and susceptible dose-dependent phenotypes were treated as susceptible in an attempt to avoid overprediction of resistance. Isolates testing intermediate and susceptible dose-dependent may have inducible genes or efflux pumps that may be challenging to identify genotypically. For some species-compound pairs, there are different interpretations from CLSI and EUCAST; as more data become available, prediction of MIC values may be possible, allowing application of different interpretative schemes. Inclusion of additional AMR markers and development of a better grasp of regulatory mechanisms and efflux pump specificity, alongside determination of how to treat co-occurring markers and discovery of new AMR markers, are needed. Finally, in this study, measurements of MICs in triplicate might have been helpful to remove uncertainty intrinsic to phenotypic susceptibility testing methods.

### Conclusion.

High concordance in species identification between the reference method, MALDI-TOF MS, and the tested WGS-based method illustrates the performance of WGS for reliable species identification. Furthermore, WGS-based prediction of AST using ARESdb demonstrates the potential of predictive AST. With continuous expansion and availability of sequenced isolates and reference AST data, together with an improved understanding of AMR, predictive AST is likely to improve and provides an opportunity for future incorporation into clinical practice.

## Supplementary Material

Supplemental file 1
