# Supplementary material for: Species Identification and Antibiotic Resistance Prediction by Analysis of Whole-Genome Sequence Data by Use of ARESdb: an Analysis of Isolates from the Unyvero Lower Respiratory Tract Infection Trial
Source: J Clin Microbiol. 2020 Jun 24;58(7):e00273-20. doi: 10.1128/JCM.00273-20 (PMC7315026; doi:10.1128/JCM.00273-20)
Supplement: Supplemental file 1 [file JCM.00273-20-s0001.pdf]

SUPPLEMENTARY FIGURES AND TABLES

Sample	Isolate	MALDI-TOF	WGS-taxonomy
**1**	**AS012628**	*Pseudomonas*	*Stenotrophomonas*
	AS012871	*Staphylococcus*	*Staphylococcus*
**2**	**AS012643**	*Stenotrophomonas*	*Pseudomonas*
	AS012644	*Stenotrophomonas*	*Stenotrophomonas*
**3**	**AS012483**	*Klebsiella*	*Providencia*
**4**	**AS012728**	*Stenotrophomonas*	*Pseudomonas*
	AS012789	*Stenotrophomonas*	*Stenotrophomonas*

Supplementary Table 1: Taxonomic identifications obtained with matrix-assisted laser desorption ionization time-of-flight mass spectrometry (MALDI-TOF) and whole genome sequencing (WGS) for the 4 isolates (bolded) discrepant at the genus level. Additional isolates that resulted from the same original patient samples are listed underneath each discrepant isolate.

**Strain ID**	**Identified By**
Acinetobacter baumannii	WGS & MALDI-TOF
Acinetobacter lactucae	WGS
Acinetobacter nosocomialis	WGS & MALDI-TOF
Acinetobacter pittii	WGS & MALDI-TOF
Citrobacter freundii	WGS & MALDI-TOF
Citrobacter koseri	WGS & MALDI-TOF
Citrobacter species FDAARGOS 156	WGS
Enterobacter asburiae	WGS & MALDI-TOF
Enterobacter cloacae	WGS & MALDI-TOF
Enterobacter hormaechei	WGS
Enterobacter kobei	MALDI-TOF
Enterobacter xiangfangensis	WGS
Escherichia coli	WGS & MALDI-TOF
Haemophilus influenzae	WGS & MALDI-TOF
Klebsiella aerogenes	WGS & MALDI-TOF
Klebsiella michiganensis	WGS
Klebsiella oxytoca	WGS & MALDI-TOF
Klebsiella pneumoniae	WGS & MALDI-TOF
Klebsiella variicola	WGS & MALDI-TOF
Moraxella catarrhalis	WGS & MALDI-TOF
Morganella morganii	WGS & MALDI-TOF
Proteus mirabilis	WGS & MALDI-TOF
Proteus vulgaris	MALDI-TOF
Providencia stuartii	WGS & MALDI-TOF
Pseudomonas aeruginosa	WGS & MALDI-TOF
Raoultella ornithinolytica	WGS & MALDI-TOF
Raoultella planticola	MALDI-TOF
Serratia marcescens	WGS & MALDI-TOF
Staphylococcus aureus	WGS & MALDI-TOF
Stenotrophomonas maltophilia	WGS & MALDI-TOF
Streptococcus mitis	WGS
Streptococcus pneumoniae	WGS & MALDI-TOF
Streptococcus salivarius	WGS & MALDI-TOF
Streptococcus vestibularis	MALDI-TOF

Supplementary Table 2: Species identified by matrix-assisted laser desorption ionization time-of-flight mass spectrometry (MALDI-TOF) or whole genome sequencing (WGS)-based identification or both. If only one method is listed, the other method did not identify the species in any of the isolates.

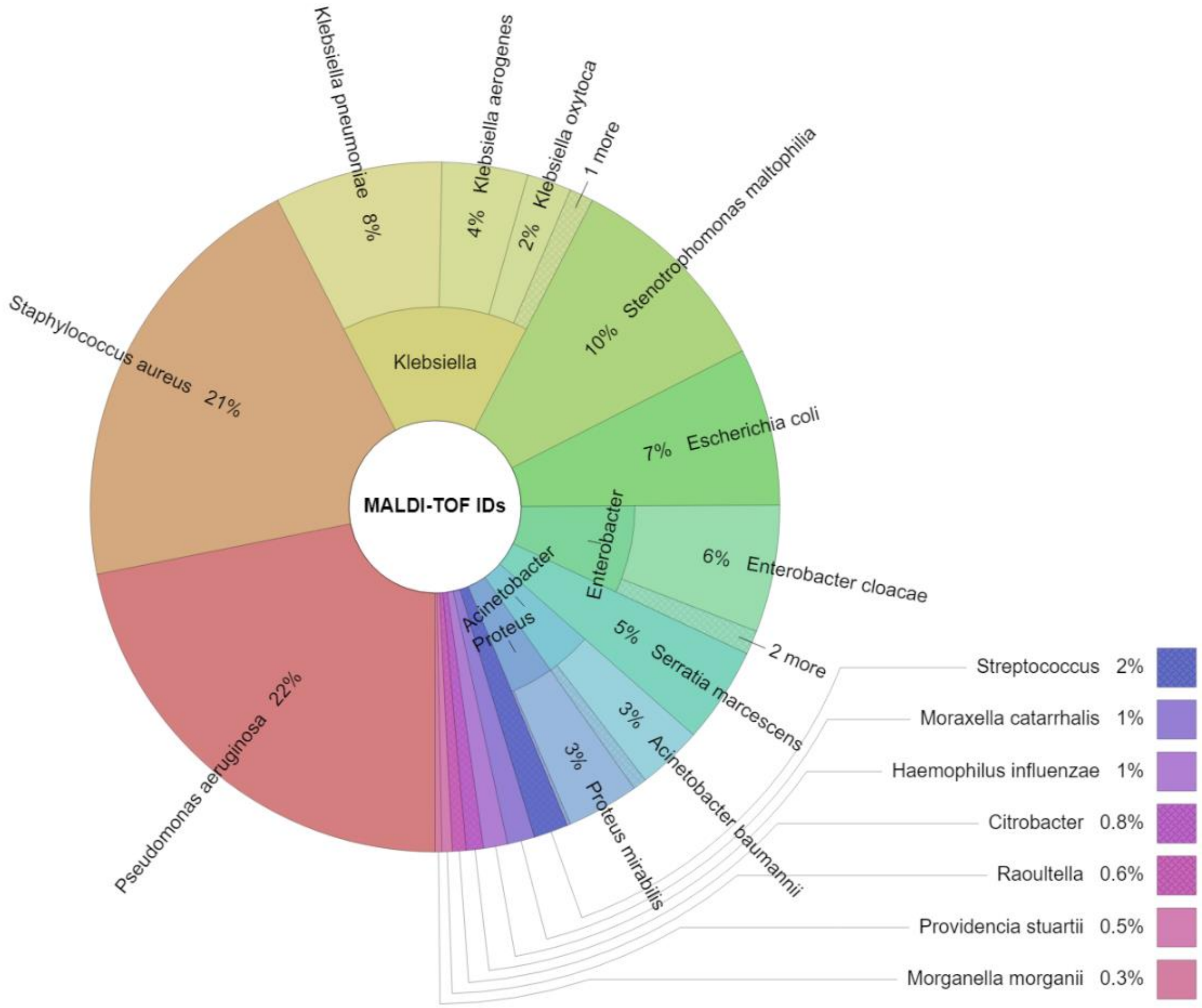

Supplementary Figure 1: Overview of pathogen identification as determined by matrix-assisted laser desorption ionization time-of-flight mass spectrometry (MALDI-TOF). The pathogen distribution is only reflective of the analyzed data set and not lower respiratory tract infection in general. Grouped species ('2 more') under the genus Enterobacter *include* Enterobacter kobei *and* Enterobacter asburiae*; grouped species under the genus* Acinetobacter *include* Acinetobacter pittii *and* Acinetobacter nosocomialis*; grouped species under the genus* Streptococcus *include* Streptococcus salivarius, Streptococcus cristatus, Streptococcus *species and* Streptococcus vestibularis. *Collapsed species represent less than 2% of the identifications*.

Compound	*Pseudomonas aeruginosa* (135)	*Stenotrophomonas maltophilia* (60)	*Klebsiella pneumoniae* (48)	*Escherichia coli* (46)	*Enterobacter cloacae* (37)	*Serratia marcescens* (28)	*Klebsiella aerogenes* (25)	*Proteus mirabilis* (21)	*Acinetobacter baumannii* (19)	*Klebsiella oxytoca* (13)	*Citrobacter freundii* (4)	*Morganella morganii* (2)
**Aminoglycosides**												
Amikacin	66.7	IR	**0.0**	ND	ND	ND	ND	ND	18.2	ND	ND	ND
Gentamicin	23.5	IR	**0.0**	**0.0**	33.3	X	ND	**0.0**	7.7	**0.0**	X	**0.0**
Tobramycin	29.4	IR	**0.0**	**0.0**	ND	**0.0**	ND	33.3	15.4	ND	X	ND
**Penicillins**												
Amoxicillin-clavulanic acid	IR	IR	43.8	66.7	IR	ND	IR	ND	IR	40.0	IR	IR
Ampicillin	IR	IR	IR	8.8	IR	ND	IR	**0.0**	IR	IR	IR	IR
Piperacillin-tazobactam	34.4	IR	X	X	X	X	X	ND	**0.0**	X	X	ND
**Cephalosporins**												
Cefuroxime	IR	IR	**0.0**	11.1	**0.0**	ND	26.7	25.0	IR	50.0	IR	IR
Ceftazidime	51.6	13.2	**0.0**	14.3	40.0	100.0	12.5	ND	**0.0**	X	50.0	**0.0**
Ceftriaxone	IR	IR	**0.0**	**0.0**	31.2	16.7	22.2	**0.0**	ND	20.0	100.0	100.0
Cefepime	61.9	9.1	**0.0**	**0.0**	**0.0**	**0.0**	ND	**0.0**	**0.0**	ND	ND	ND
**Fluoroquinolones**												
Ciprofloxacin	10.0	ND	10.0	**0.0**	40.0	**0.0**	50.0	**0.0**	**0.0**	50.0	X	**0.0**
Levofloxacin	21.6	66.7	22.2	6.2	50.0	**0.0**	100.0	**0.0**	**0.0**	**0.0**	X	**0.0**
**Carbapenems**												
Ertapenem	IR	IR	**0.0**	100.0	80.0	100.0	50.0	ND	IR	ND	IR	ND
Imipenem	22.7	IR	**0.0**	X	ND	50.0	**0.0**	50.0	**0.0**	ND	ND	100.0
Meropenem	21.1	IR	**0.0**	X	ND	X	ND	ND	**0.0**	ND	ND	ND
**Folate pathway inhibitor**												
Trimethoprim-sulfamethoxazole	ND	94.4	18.8	33.3	16.7	75.0	66.7	14.3	**0.0**	X	100.0	**0.0**

Pathogen

Pathogen	Gentamicin	Benzylpenicillin	Ciprofloxacin	Levofloxacin	Moxifloxacin	Erythromycin	Tetracycline	Trimethoprim	Trimethoprim-sulfamethoxazole
Staphylococcus aureus (128)	50.0	3.3	**0.0**	**0.0**	**0.0**	37.7	40.0	11.1	50.0

Compound

Supplementary figure 2: Very major errors (VME) per species-compound pair, with results for Gram-negative bacteria shown on the top and Staphylococcus aureus on the bottom. Pairs where species are considered intrinsically resistant to the compound are labelled as "IR". Species-compound pairs not tested by phenotypic susceptibility testing or without genotypic susceptibility prediction are labelled with "ND". Species-compound pairs without resistant samples, for which VMEs cannot be calculated are labelled with "X". Sample counts per species are listed in parentheses. VMEs below 1.5% are bolded.

Compound	*Pseudomonas aeruginosa* (135)	*Stenotrophomonas maltophilia* (60)	*Klebsiella pneumoniae* (48)	*Escherichia coli* (46)	*Enterobacter cloacae* (37)	*Serratia marcescens* (28)	*Klebsiella aerogenes* (25)	*Proteus mirabilis* (21)	*Acinetobacter baumannii* (19)	*Klebsiella oxytoca* (13)	*Citrobacter freundii* (4)	*Morganella morganii* (2)
**Aminoglycosides**												
Amikacin	**0.8**	IR	**0.0**	ND	ND	ND	ND	ND	**0.0**	ND	ND	ND
Gentamicin	5.1	IR	**0.0**	**0.0**	**0.0**	7.1	ND	5.6	16.7	**0.0**	**0.0**	**0.0**
Tobramycin	4.2	IR	**2.5**	7.0	ND	3.8	ND	**0.0**	**0.0**	ND	25.0	ND
**Penicillins**												
Amoxicillin-clavulanic acid	IR	IR	3.1	**0.0**	IR	ND	IR	ND	IR	**0.0**	IR	IR
Ampicillin	IR	IR	IR	8.3	IR	ND	IR	**0.0**	IR	IR	IR	IR
Piperacillin-tazobactam	20.4	IR	16.7	**0.0**	21.6	3.6	12.0	ND	**0.0**	38.5	25.0	ND
**Cephalosporins**												
Cefuroxime	IR	IR	15.8	**0.0**	76.9	ND	60.0	11.8	IR	14.3	IR	IR
Ceftazidime	10.6	22.7	**0.0**	5.1	22.7	**0.0**	47.1	ND	**0.0**	7.7	**0.0**	**0.0**
Ceftriaxone	IR	IR	**0.0**	**0.0**	33.3	**0.0**	50.0	5.6	ND	**0.0**	**0.0**	**0.0**
Cefepime	14.0	40.0	**2.5**	**2.4**	5.6	**0.0**	ND	10.5	37.5	ND	ND	ND
**Fluoroquinolones**												
Ciprofloxacin	9.5	ND	5.3	**0.0**	**0.0**	16.0	4.3	**0.0**	**0.0**	**0.0**	**0.0**	X
Levofloxacin	10.7	8.3	**0.0**	**0.0**	6.1	7.7	8.3	**0.0**	**0.0**	**0.0**	**0.0**	X
**Carbapenems**												
Ertapenem	IR	IR	8.7	**2.3**	6.2	**0.0**	47.6	ND	IR	ND	IR	ND
Imipenem	15.4	IR	**0.0**	**0.0**	ND	3.8	**0.0**	41.2	**0.0**	ND	ND	**0.0**
Meropenem	21.6	IR	15.2	4.3	ND	**0.0**	ND	ND	**0.0**	ND	ND	ND
**Folate pathway inhibitor**												
Trimethoprim-sulfamethoxazole	ND	**2.4**	**0.0**	3.6	**0.0**	**0.0**	4.5	**0.0**	20.0	15.4	**0.0**	X

Pathogen

Pathogen	Gentamicin	Benzylpenicillin	Ciprofloxacin	Levofloxacin	Moxifloxacin	Erythromycin	Tetracycline	Trimethoprim	Trimethoprim-sulfamethoxazole
Staphylococcus aureus (128)	**0.0**	11.8	**1.8**	5.3	5.2	10.0	8.1	**0.8**	22.5

Compound

Supplementary figure 3: Major error (ME) rates per species-compound pair, with results for Gram-negative bacteria shown on the top and those for Staphylococcus aureus on the bottom. Pairs where species are considered intrinsically resistant to the compound are labelled as "IR". Species-compound pairs not tested by phenotypic susceptibility testing or without genotypic susceptibility prediction are labelled with "ND". Species-compound pairs without susceptible samples for which MEs cannot be calculated are labelled with "X". Sample counts per species are listed in parentheses. MEs below 3.5% are bolded.
